# Isolation, identification and characterization of *Streptomyces* metabolites as a potential bioherbicide

**DOI:** 10.1371/journal.pone.0222933

**Published:** 2019-09-23

**Authors:** Aung B. Bo, Jae D. Kim, Young S. Kim, Hun T. Sin, Hye J. Kim, Botir Khaitov, Young K. Ko, Kee W. Park, Jung S. Choi

**Affiliations:** 1 Department of Crop Science, Chungnam National University, Daejeon, Korea; 2 Eco-friendly and New Materials Research Group, Korea Research Institute of Chemical Technology, Daejeon, Korea; Dongguk University, REPUBLIC OF KOREA

## Abstract

Bioactive herbicidal compounds produced by soil microorganisms might be used to creating a bioherbicide for biological weed control. A total of 1,300 bacterial strains were isolated and screened for herbicidal activity against grass and broadleaf weeds. Among primarily selected 102 strains, the herbicidal activity of bacterial fermentation broths from the following three isolates strain-101, strain-128, and strain-329 reduced the growth of *D*. *sanguinalis* by 66.7%, 78.3%, and 100%, respectively as compared with control. Phylogenetic analysis of 16S rRNA gene sequencing determined that the strain-329 has 99% similarity to *Streptomyces anulatus* (HBUM 174206). The potential bioherbicidal efficacy of *Streptomyces* strain-329 was tested on grass and broadleaf weeds for phytotoxic activity through pre- and post-emergence applications. At pre-emergence application, the phytotoxic efficacy to *D*. *sanguinalis* and *S*. *bicolor* on seed germination were 90.4% and 81.3%, respectively at the 2x concentration, whereas in the case of *Solanum nigrum*, 85.2% phytotoxic efficacy was observed at the 4x concentration. The efficacy of *Streptomyces* strain-329 was substantially higher at post-emergence application, presenting 100% control of grass and broadleaf weeds at the 1x concentration. Two herbicidal compounds coded as 329-C1 and 329-C3 were extracted and purified by column chromatography and high-performance liquid chromatography methods. The active compound 329-C3 slightly increased leaf electrolytic leakage and MDA production as concentration-dependent manner. These results suggest that new *Streptomyces* sp. strain-329 produced bioherbicidal metabolites and may provide a new lead molecule for production an efficient bioherbicide to regulate grass and broadleaf weeds.

## Introduction

Weeds are the main problem in crop production, causing substantial losses to the yield. Continuous use of synthetic herbicides for weed control exacerbates long-term ecological challenges such as environmental pollutions, harmful herbicide residue accumulation in soil and water resources, mammalian toxicity, and the evolution of herbicide resistance in prevalent weed species [1, 2]. Current concern on the environmental protection made it essential to develop safe, non-toxic, and eco-friendly bioherbicides in order to decrease of chemical input into the ecosystem [3].

Nowadays, researches on the development of bioherbicides and their phytotoxic metabolites have been expanded in many countries that may play a major role towards sustainability in agriculture [4]. Bioherbicides are compounds that derived from microorganisms such as fungi, bacteria, viruses and also some plant species. Several researchers declared that naturally derived bioherbicides are effective substances for weed control, thereby provide ecological advantages and maintain sustainable agricultural production [5, 6]. Secondary metabolites produced by these species cause plant phytotoxic activities such as necrosis, chlorosis, deformation and stunting[7]. These features are prerequisite to use them as bioherbicides for weed control [8]. Furthermore, more effective compounds can be found by deeper exploring the quantitative structure of active metabolites generated by microorganisms [9]. Therefore, since the 1990s, scientists have focused on bacterial herbicides to control weeds [10]. However, weak herbicidal activity is the main constrains towards the development and large-scale use of bioherbicides [11, 12]. However, modern techniques can enhance herbicidal activity of bioherbicides through synthetic modifications and additional adjuvants [13]. Recent findings revealed that phytotoxic metabolites vary in their degree of target-specificity, though weed species may also differ in susceptibility to a particular toxin [14].

Some soil microorganisms can produce valuable secondary metabolites including bioactive herbicidal compounds that might be used to create as a good candidate for biological weed control [15]. *Streptomyces* are a major group of inhabitant phytopathogenic bacteria with herbicidal activity living in soil [16]. For example, Bilanaphos has been commercialized as a herbicide in 1983, which constituted the extracts of *Streptomyces hygroscopicus* SF1293 [17]. Moreover, glufosinate ammonium formulated with the metabolites produced by *Streptomyces hygroscopicus* which was successfully commercialized in 1984, and currently it is at the top of 10 bioherbicides which have been using all around the world [18, 19]. Blasticidin S constitutes the extracts of *Streptomyces griseohromogenes*, herbicidans A and B consist of *Streptomyces saganonensis* and *Streptomyces scopuliridis*; and nigericin, hydantocidin and geldanamycin were developed as bioherbicides from the active compounds of *Streptomyces hygroscopicus* [16, 12].

However, phytotoxic activity of *Streptomyces* as a target-specific bioherbicide was not thoroughly studied in Korea. Therefore, the present study was conducted at two stages: (1) isolation of *Streptomyces* from different sites of Korea and screening for their phytotoxic activity against common broadleaf and grass weeds; (2) determination of chemical formulation and purification of new herbicidal active compounds derived from secondary metabolites of selected *Streptomyces* strains.

## Materials and methods

### Isolation of Streptomyces

A total of 1,300 soil samples were collected from 10–20 cm depth of forests, non-agricultural lands, and coastal areas of Cheongtaesan Recreation Forest located in Cheongtaesan-ro, Dunnae-myeson, Hoengseong-gun, Gangwon-do, Republic of Korea. Slightly acidic podzolic soil with pH 5.7–6.6 and EC 1.2–2.4 dS/m widely distributed all around the soil collection area. This type of soil consists 545–590 g kg-1 of clay, 255–290 g kg-1 of silt, 17.8–26.1 g kg-1 of organic matter, and 148–171 g kg-1 of sand. These soil samples were prepared for isolation of bacterial strains by the standard serial dilution method [20]. One gram of each sample was suspended in 9 ml of distilled water and vortexed. Then, serial dilutions of each sample were carried out up to 10^−3^ dilution.

Isolation of *Streptomyces* was conducted by the procedure of Hayakawa and Nonomura [21]. The 100 μL of each aliquot from final dilutions was spread over the surface of Humic acid-Vitamin agar containing humic acid dissolved in bacteriological agar 20 g, MgSO_4_ 0.5 g, Na_2_HPO_4_ 5 g, CaCO_3_ 0.2 g, humic acid 10 g, KCL 20 g, cycloheximide 5 g, distilled water 1 L. The cultured plates were incubated at 25°C in darkness until sporulations of bacterial colonies for two weeks. Bacterial colonies were identified on the basis of morphological characteristics by light microscopy. Representative colonies of *Streptomyces* were picked up and streaked on fresh Bennet’s agar plates. The composition of Bennet’s media contains glucose 10 g, yeast extract 1 g, peptone 2 g, beef extract 1 g, bacteriological agar 20 g, and distilled water 1 L. These streaking plates were incubated at 25°C and monitored after seven days. Repeated streaking on Bennet’s agar medium led to the purification of bacterial colonies. Pure and single colonies of *Streptomyces* were picked and preserved at 2–3°C for further evaluation of herbicidal activity.

### PCR amplification of 16S rRNA

The *Streptomyces* strain which had the highest herbicidal activity was characterized by through16S rRNA gene sequencing. PCR amplification was performed using primers 27F (5'-AGAGRR TGARCCTGGCTCAG-3') and 1492R (5'-GGR TACCTTGRRACGACTT-3'). The genomic DNA was extracted from pure broth liquid with DNeasy Blood & Tissue kit (Qiagen, Hilden, Germany). For obtaining DNA, a single colony of bacterial cell was disrupted by heating at 100°C for 10 min. Then, for spore-forming isolate, the bacterial cell was disrupted in a microwave at 650 W for 30 s in a buffer. The suspensions were centrifuged at 13,000 rpm for 10 min. After centrifugation, 2 μl of the supernatants were used for PCR reactions. PCR products were purified and sequenced at SolGent Inc. The sequence data was submitted to the GenBank for BLAST analysis. Multiple sequence alignment and molecular phylogenetic analysis were performed using BioEdit and MEGA version 4.0 software packages. The phylogenetic tree was constructed using the Neighbor-joining method (PHYDIT program version 3.0) [22].

### Screening for herbicidal activity

The isolated *Streptomyces* strains were maintained as spore suspensions and mycelial fragments in 25% glycerol (v/v) at -70°C in a deep freezer. These strains were cultured in M3 (soytone 10 g, glucose 10 g, soluble-starch 20 g, CaCO_3_ 3 g, and distilled water 1 L) liquid medium and incubated at 27°C and 160 rpm for a week. When spores reached 10^7^ L^-1^ CFU (colony forming unit), the biomass of bacteria was harvested by centrifuging of fermentation broth for 10 min (8,000 rpm). The solutions were prepared by traditionally serial dilutions of culture liquid with distilled water at the ratio of 1 ml of culture liquid and 9 ml distilled water for 1x concentration. Then, 5 ml of the fermentation medium at the 1x concentration was sprayed on the foliage of *Digitaria sanguinalis* to evaluate herbicidal effect. In all experiments, 0.1% of Tween 20 was added to reduce surface tension of the supernatant. Herbicidal activities were monitored visually seven days after treatment (DAT) on the basis physiological and morphological symptoms of mortality (0 = no activity, 10–30 = slight activity, 40–60 = moderate activity, 70–90 = strong activity, and 100 = complete death) by the mean of percent in three replicates (twenty plants per pot) [23].

### Bio-herbicidal assay of *Streptomyces*

Seeds of common grass weeds (*Digitaria sanguinalis*, *Sorghum bicolor*, *Echinochloa crus-galli*, *Agropyron smithii*) and broadleaf weeds (*Solanum nigrum*, *Aeschynomene indica*, *Xanthium strumarium*, and *Calystegia japonica*) (20 seeds per pot with three replications) were sown in 6cm pots containing commercial horticultural soil under greenhouse (25/15±5°C, Light/Dark = 12/12 h). The concentrations of isolated *Streptomyces* strains were 1x, 2x, and 4x for pre-emergence application, and 1/4x, 1/2x, and 1x for post-emergence application. The treatments were randomized in three replications with 20 seeds per pot/replication of each weed species. The seeds were previously surface-sterilized with a solution of 75 ml 0.1% mercuric chloride + 25 ml water for 5 min and rinsed few times with distilled water. For pre-emergence, each fermentation medium with a volume of 50 ml was sprayed to the soil in a pot immediately after planting the seeds to evaluate its bioherbicidal activity in the bioassay. Distilled water was used as the control. The experiment was conducted in a completely randomized design with three replications. The pots later incubated in a chamber at 25°C under a photoperiod of 12 h for 7 days. The effect of phytotoxic activity was evaluated 14 DAT for pre-emergence application and 7 DAT for post-emergence application. Germination percentage of the seeds was assessed for each replication by the equation:

$$G=(\frac{Germinated\;seed}{Total\;seed}*100)$$

### Purification and structural identification of herbicidal compound

The purification and structural identification of *Streptomyces* active strain were carried out according to the method described by Lee *et al*. [23]. The microbial active strain coded as *Streptomyces* strain-329 was cultured in M3 liquid medium for 7 days at 27°C and then the culture broth was centrifuged at 6000 rpm to remove cell debris. The filtrate of fermentation broth was extracted with hexane, chloroform, ethyl acetate, and butanol, and concentrated under reduced pressure concentrator (EYELA SB-1200, EYELA Shanghai Co., Ltd) and separated by silica gel column chromatography using a chloroform/methanol solvent system (4:1 v/v). The herbicidal activity of fractions was purified by series of Sephadex LH-20 column chromatography and eluted with methanol. Further, the active fractions were successively separated by Sephadex G-10 column chromatography and eluted with H_2_O. Each activity was determined through seed germination of *Digitaria sanguinalis*. The active fractions were purified by preparative high-performance liquid chromatography (HPLC). The preparative HPLC was conducted on JASCO HPLC (PU890, MD910, Tokyo, Japan), analytical HPLC on Hewlett-Packard 1090 Type II with LiChrospher RP18 (150 × 10 mm) and an H_2_O/MeOH gradient.

The chemical structures of herbicidal compounds were elucidated by ^1^H- and ^13^C-NMR and mass spectra ESI-MS (YL9100S, Young Lin device). ESI-MS was recorded by VG Quattro 440 mass. ^1^H- and ^13^C-NMR spectra were recorded on AC 300 and DMS 600 (BRUCKER, Karlsruhe, Germany) with the chemical shifts being represented as part per million (ppm) referenced to CD_3_OD signal as solvent (3.30 ppm at ^1^H- and 49.0 ppm at ^13^C-NMR spectrum).

#### Evaluation of herbicidal activity of herbicidal compound on weed growth

Seeds of grass species, *D*. *sanguiinalis*, *Sorghum bicolor*, *Echinochlia crus-galli*, *Agropyron smithii* and broad leaf species, *Solanum nigrum*, *Aeschynomene indica*, *Xanthium strumarium*, *Calystegia japonica* were germinated in a greenhouse at 30/20 ± 5°C day/night temperature under 14/10 h light/dark condition. Fourteen-day old seedlings were grown in pots and assessed by post-emergence test with *Streptomyces* herbicidal compounds and Cycloheximide which were 60S of the 80S ribosomes in a eukaryotic cell representing a strong antimicrobial activity. Cycloheximide has been isolated from the culture filtrate *Streptomyces griseus* as the active ingredient of fungicidal product against leaf blights and rust disease. The phytotoxic effects of Cycloheximide (naramycin A) are well known. It acts as an abscission agent and is applied to orange, grapefruit, and olive cultures. For that reason, practical application as fungicide is only possible in a few indications.

These compounds were diluted up to 62.5, 125, 250, 500 μg ml^-1^ containing 0.1% Tween-20 and acetone 50% and then 14 ml was sprayed directly to the leaves and stems of the tested plants. The herbicidal activities (control values) were evaluated visually 7 days after treatment on the basis of morphological and physiological symptoms according to the mean percentage score of three replicates of mortality (0, no activity; 10–30, slight activity; 40–60, moderate activity; 70–90, strong activity; 100, complete death).

### Chlorophyll determination

Fully expanded cucumber plants were treated with herbicidal compound of *Streptomyce* KRA14-329, glufosinate, and paraquat for assessment of chlorophyll content. Cucumber (*Cucumis sativus*) leaf disks (8 mm diameter and 1 g fresh weight) were transferred into a 15 ml test tube containing 7 ml of distilled water. After vacuum infiltration at 50 kPa for 20 min, the water was removed and replaced with treatment solutions. The leaf disks were incubated in a growth chamber at 25°C under darkness for 12 h and then exposed to continuous white light at 120 μmol m^-2^ sec^-1^ photosynthetically active radiation for 24 h. After incubation, chlorophyll was extracted and assayed according to the procedure of Hiscox and Israelstam [24]. The leaf disks were soaked under darkness in 10 ml of dimethyl sulfoxide (DMSO) for 24 h at room temperature. Total chlorophyll content of the extracts was determined spectrophotometrically. The tests were replicated three times. This method was also used to measure injuries (chlorosis and bleaching) in the leaf disc bioassays caused by exposure of *Streptomyces*, glufosinate, and paraquat at various concentrations. Total chlorophyll contents were determined by this formulation;

Chlorophyll(mgL)=(20.2*A645nm+8.02*A663nm)

### Cellular leakage

Cellular leakage assay was performed in the leaf tissues of 7 days old *Crocus sativus* seedlings. A total of 50 leaf disks 5 mm diameter each were placed in a petri dish containing 7 ml of herbicidal compound of *Streptomyces* KRA14-329, glufosinate, and paraquat, respectively at different concentrations. The petri dishes were placed in a growth chamber under 25°C for incubation. The cellular leakage amount was determined at 12 hours after application using an electronic conductivity meter (Denki Kagaku Co., Ltd., Musashino, Japan).

### Lipid peroxidation

Lipid peroxidation was estimated by the level of malondialdehyde (MDA) production using a slight modification of thiobarbituric acid (TBA) method as previously described (Slater 1984). *C*. *sativus* leaf disks were placed in the solution of herbicidal compound of *Streptomyces* KRA14-329, glufosinate, and paraquat and incubated as the same manner as used for the measurement of chlorophyll. After incubation, the leaf disks were homogenized with a mortar and pestle in 5 ml solution of 0.5% TBA in 20% trichloroacetic acid. Then, the homogenate was centrifuged at 15,000 xg for 15 min and the supernatants were collected. The supernatants were heated in a water bath at 95°C for 25 min and allowed to cool in an ice bath. Following centrifugation at 20,000 x for 15 min, the resulting supernatants were used for spectrophotometric determination of MDA.

The aliquots of the bathing medium in which leaf disks were incubated with the different concentrations of treatment solutions were also subjected to the same procedure used for the leaf disks. Absorbance at 532 nm for each sample was recorded and corrected for non-specific turbidity at 600 nm. MDA concentration was calculated using a molar extinction coefficient of 156mM^-1^ cm^-1^ [25]. The MDA concentrations on fresh weight basis from both fractions of the tissues and the bathing medium were pooled and then regarded as total MDA produced by the leaf disks.

### Statistics

Data of repeated experiments with three replications were pooled because there was no significant difference. All data were analyzed using one way Anova. Significant differences were detected by the F-test, means were separated with student test at the 0.05 LSD level of probability.

The conducted research had no any harmful effect for humans or other living creatures, therefore, there was no need to get a special permission to conduct this experiment.

## Results

### Screening *Streptomyces* isolates with herbicidal activity

**Fig 1:**
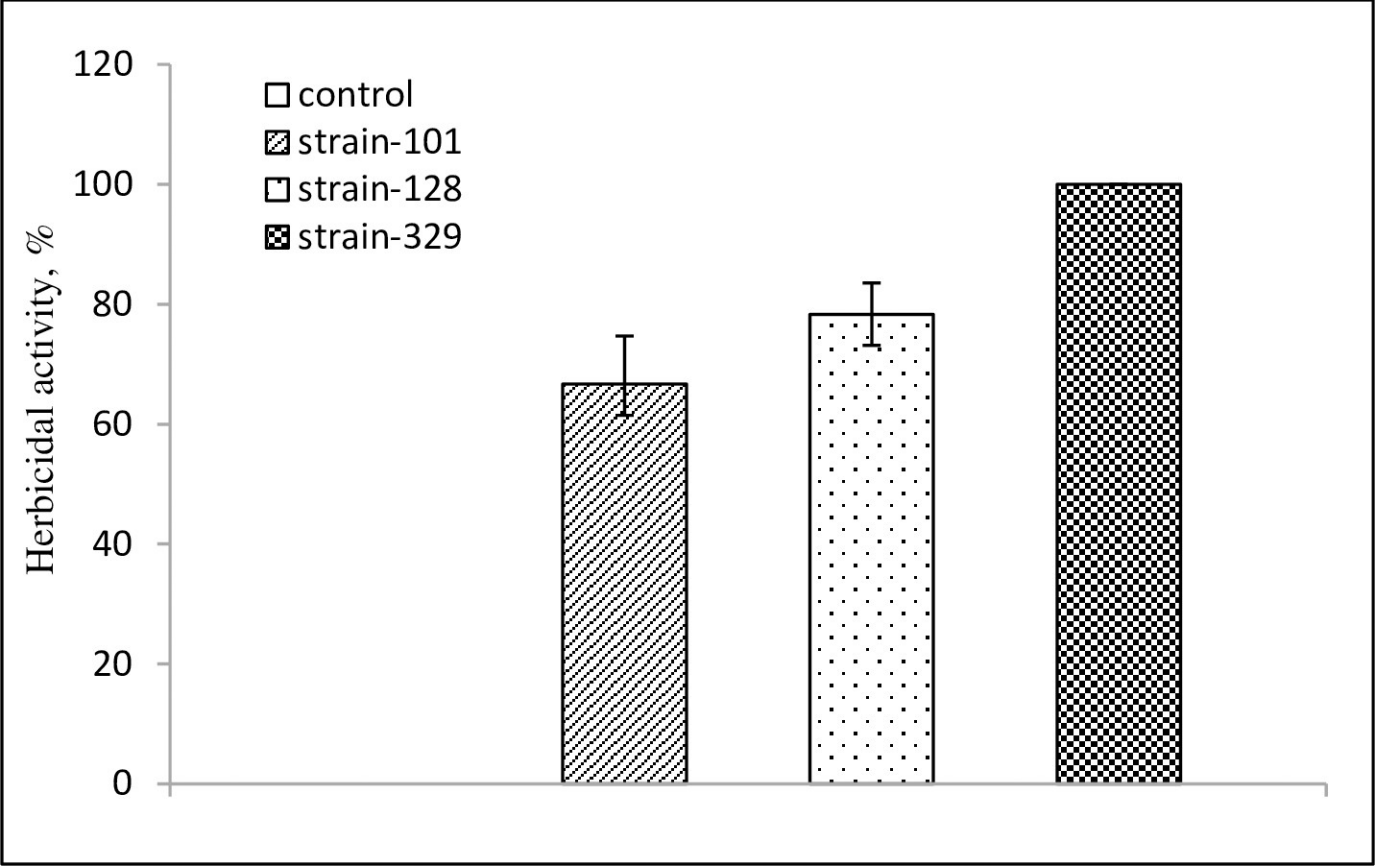
Herbicidal activity of bacterial strains on growth of *D*. *sanguinalis*.

In this experiment, 102 isolates (8%) out of 1,300 bacterial strains showed herbicide activity on the seedling growth of *D*. *sanguinalis*. Amongst the 102 isolated strains, only three isolates namely strain-101, strain-128, and strain-329 exhibited considerable herbicidal activity against *D*. *sanguinalis* (Fig 1).

The host range test conducted on these selected three isolates showed that strain-329 reduced growth with severe lesions and serious injury such as yellowing, leaf spots, and blight on the foliage of *D*. *sanguinalis*, which eventually led to the plant death. The observation of strain-101 and strain-128 revealed some lesions with low severity, but did not completely inhibit the plant growth.

As estimated, plant growth of *D*. *sanguinalis* was reduced by 66.7% and 78.3% after the application of strain-101 and strain-128, respectively, whereas strain-329 reduced plant growth of *D*. *sanguinalis* by 100% at 7 DAT. Taking into account substantial phytotoxic effect and affected morphology of the target weeds, strain-329 was selected for molecular identification and further chemical investigation to determine the optimal concentration as a potential bioherbicide.

### 16S rRNA gene amplification for *Streptomyces strain-329*

PCR based molecular methods namely 16S rRNA, RAPD and STRR can be utilized at taxonomic levels (Dhanasekaran et al. 2012). However, sequencing of genes encoding 16S rRNA is the most promising technique for phylogenetic classification of bacteria.

In this investigation, the 16S rRNA gene amplification of *Streptomyces* strain-329 was performed by the PCR technique with universal primers. A PCR product was analyzed in 1% of agarose gel. The amplified 16S rRNA sample was performed for partial sequencing of nucleotide. A BLAST of the obtained nucleotide of sequence for the *Streptomyces* strain-329 was performed and the sequence described similarity with the *Streptomyces* sp.

**Fig 2:**
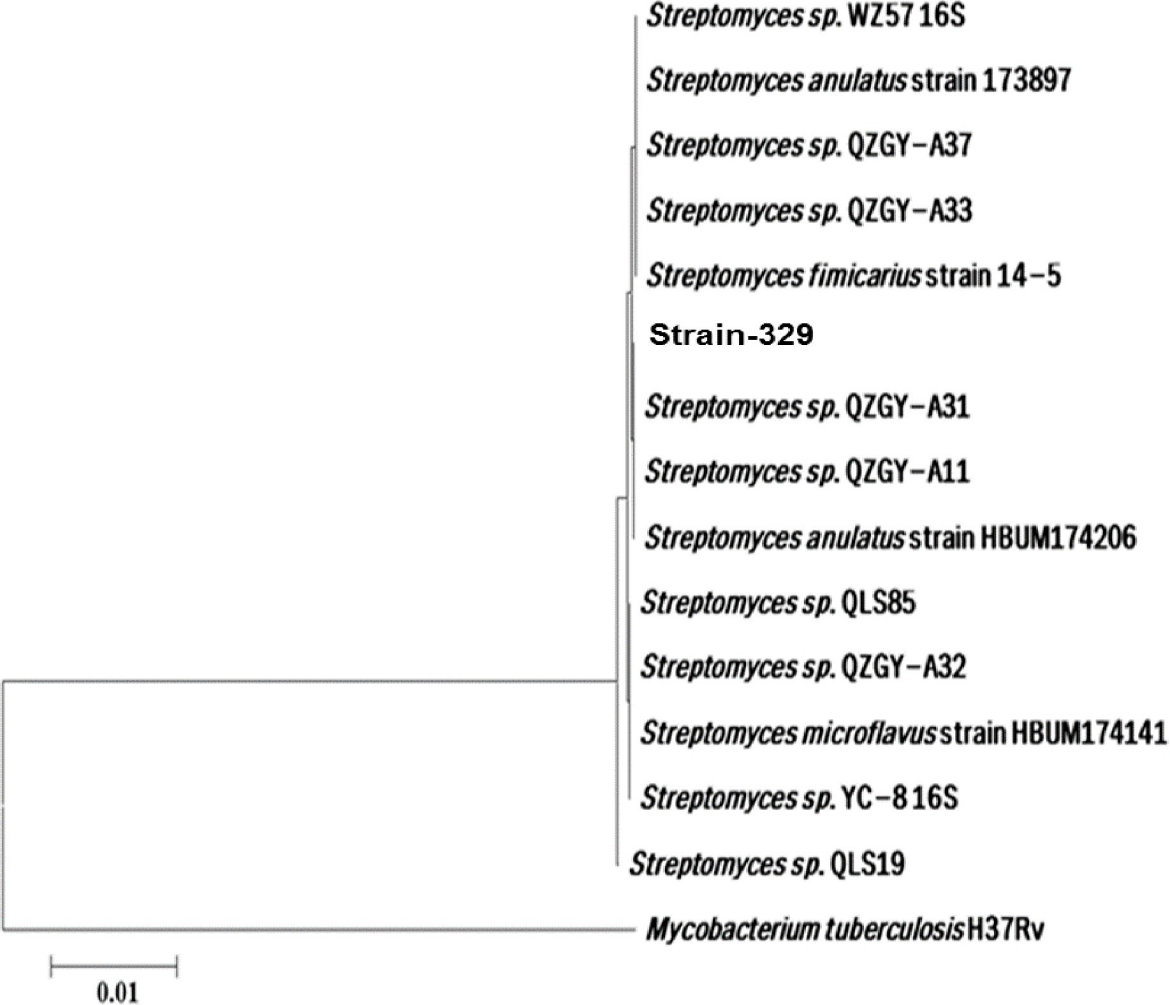
Neighbour-joining tree based on 16S rRNA gene sequences of *Streptomyces* strain-329. The evolutionary history was inferred using the Neighbor-Joining method.

The phylogenetic analysis determines evolutionary relationship between the micro-organisms [4]. Therefore, phylogenetic analysis of *Streptomyces* strain-329 was constructed based on the neighbor-joining tree using its 16S rRNA sequence with that of other *Streptomyces* sp. from the NCBI database. The data of nucleotide sequence has been set down at GenBank under accession numbers HBUM 174206. The percentage similarity was 99% (Fig 2). Based on the BLAST results, the isolated strain-329 was found to belong to *Streptomyces anulatus*. Therefore, *Streptomyces* strain-329 can be classified into the subgenus of *S*. *anulatus*.

The optimal tree with the sum of branch length = 0.17455560 is shown. The tree is drawn to scale, with branch lengths in the same units as those of the evolutionary distances used to infer the phylogenetic tree.

## Phytotoxicity of *Streptomyces* strain-329

**Fig 3:**
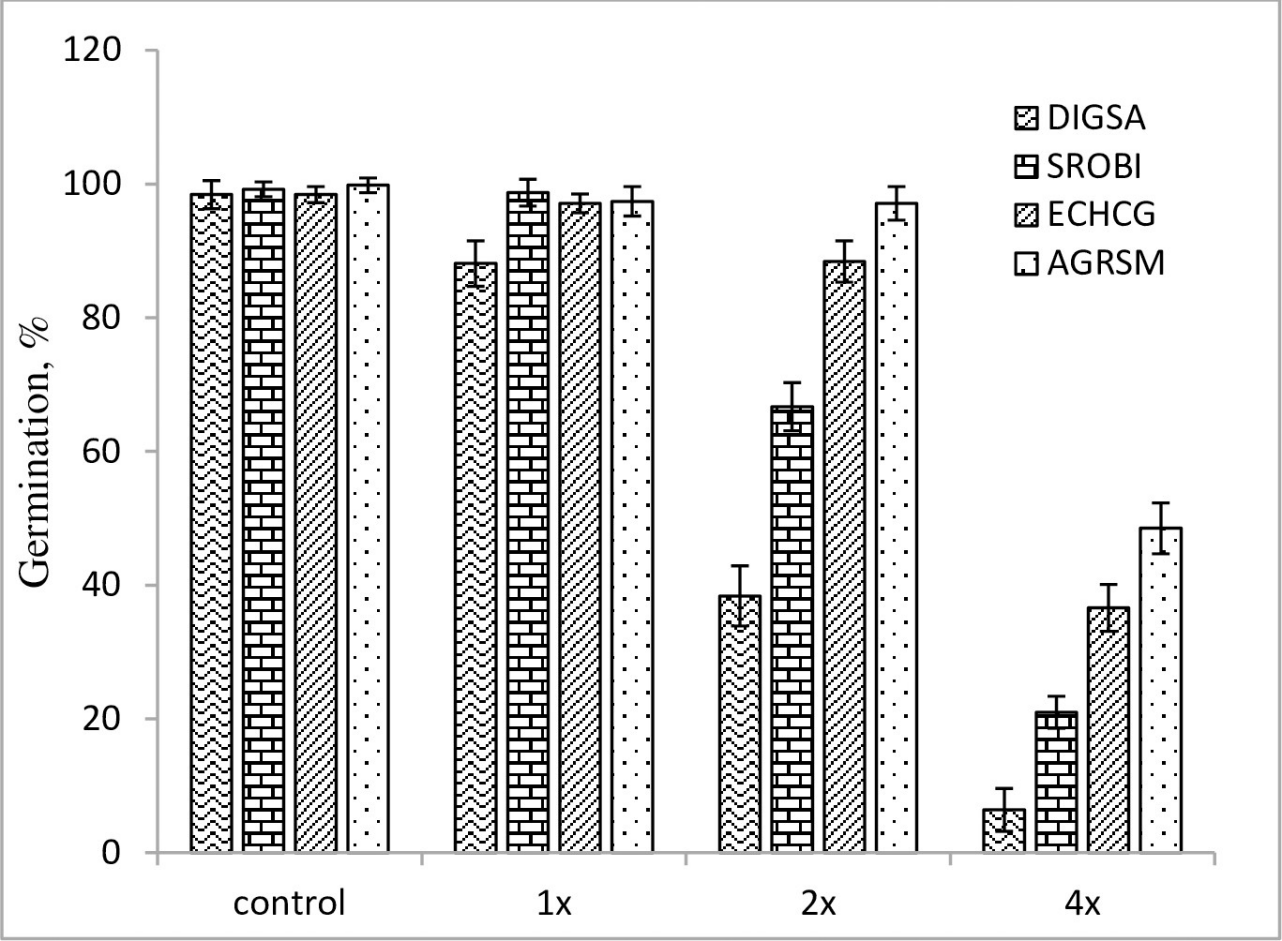
Herbicidal effect of *Streptomyces anulatus* strain-329 on germination of grass weeds in pre-emergence application at 14 days after treatment under greenhouse condition.

**Fig 4:**
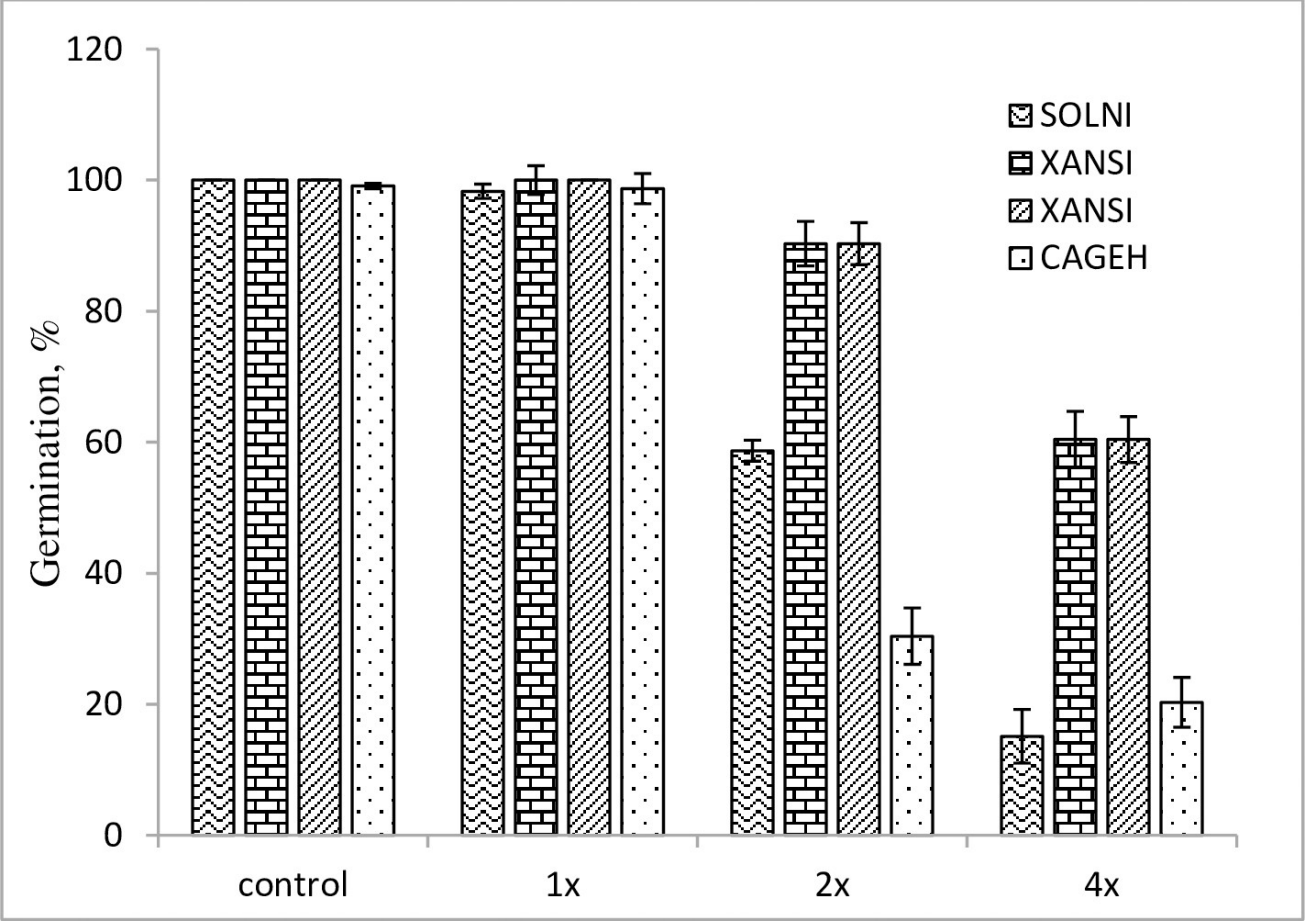
Herbicidal effect of *Streptomyces anulatus* strain-329 on germination of broadleafweeds in pre-emergence application at 14 days after treatment under greenhouse condition.

At the pre-emergence application of *Streptomyces* strain-329, the germination percentage significantly reduced in all the tested weed species, except at the 1x rate concentration (Figs 3 and 4).

Herbicidal activity of strain-329 increased significantly with increasing concentration level. The highest 4x concentration of strain-329 drastically inhibited the germination of *D*. *sanguinalis* and *S*. *bicolor* seeds by 90.4% and 81.3%, respectively than that of the control and showed a great inhibition effect. Among representatives of broadleaf weeds, *S*. *nigrum* showed a decrease of the germination rate by 85.2% as compared with control at the 4x concentration of strain-329.

The *Streptomyces* strain-329 strain showed apparent herbicidal effects on germination of the weeds, and the most remarkable result was found at the 4x concentration, exhibiting a substantial germination inhibition in the tested weeds. The bacterial strain demonstrated considerable phytotoxicity and affected morphology of the weeds.

### Leaf necrosis bioassay

**Fig 5:**
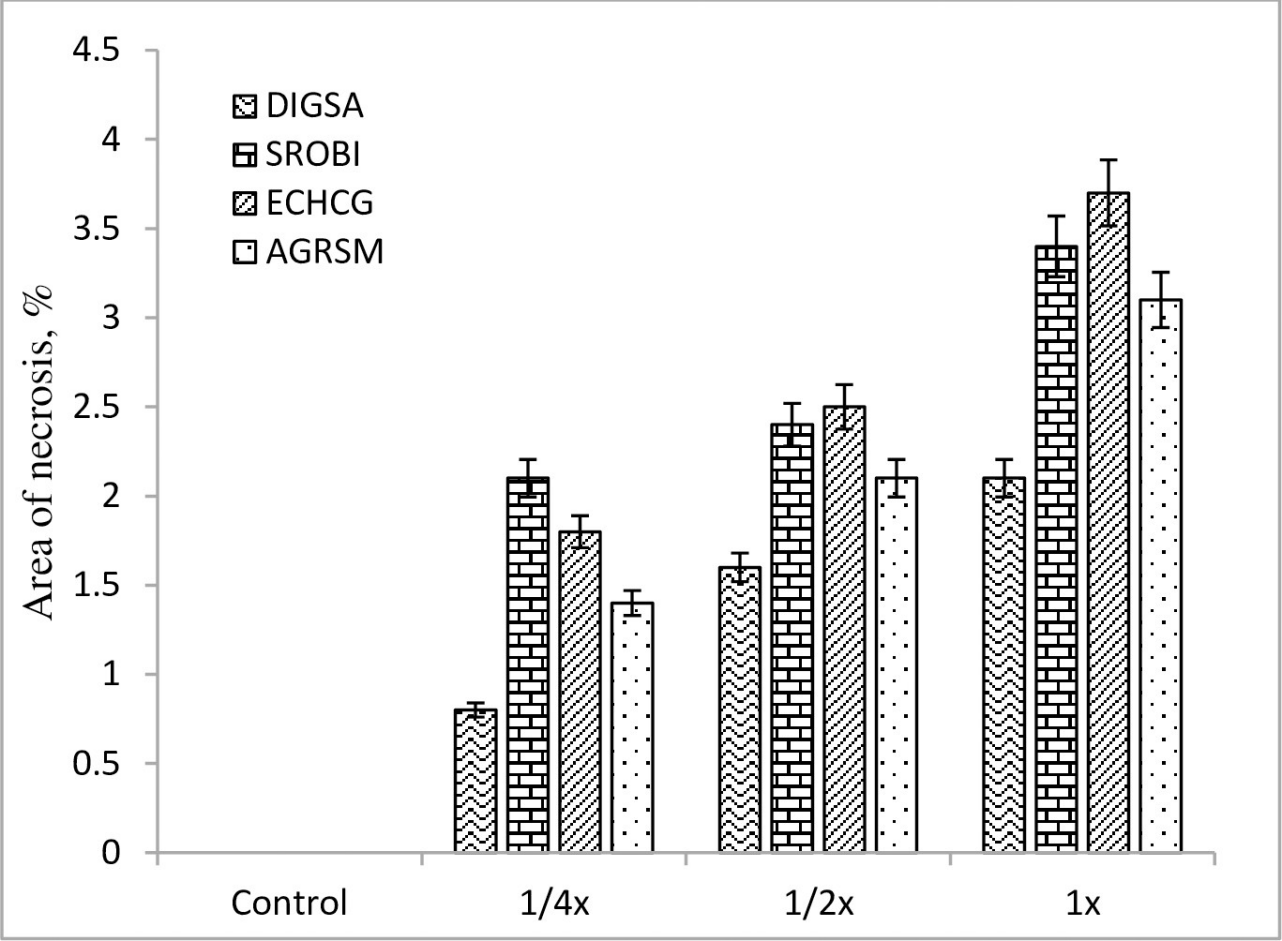
Area of necrosis in grass weeds leaves at 7 days after foliar application under greenhouse condition.

**Fig 6:**
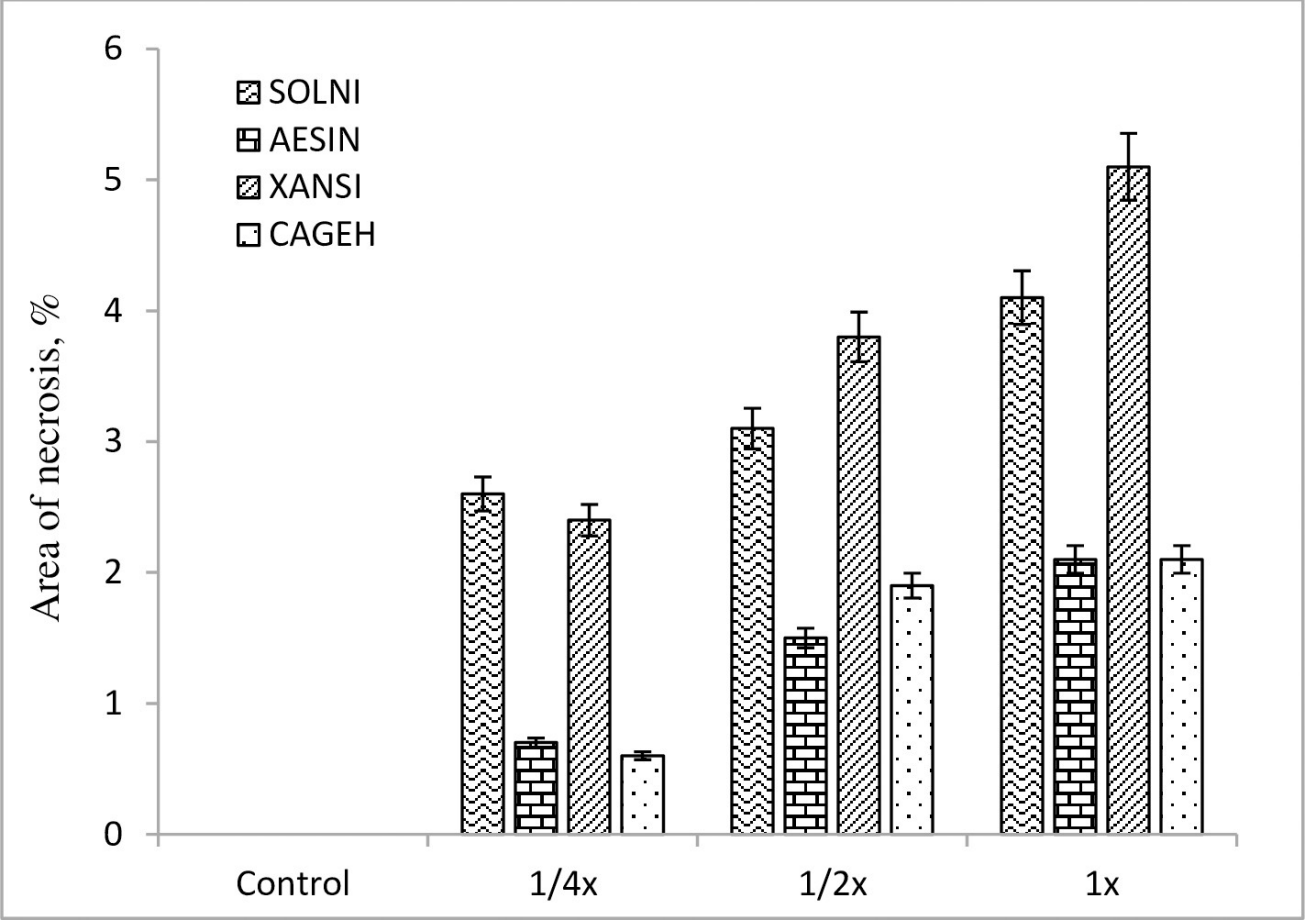
Area of necrosis in broadleafweed leaves at 7 days after foliar application under greenhouse condition.

*Streptomyces* strain-329 was able to induce a necrotic lesion on the leaves of grass and broad leaf weeds without any necrotic activity observed for the control (P ≤ 0.05). The weed species differed significantly in lesion areas, especially, the differences were obvious between grass and broadleaf weeds (Figs 5 and 6). The phytotoxic effect of strain-329 at the 1/4x concentration was inefficient, exhibiting not any lesions on grass weeds namely *Sorghum bicolor* and *Echinochloa crus-galli*, and on broadleaf weeds including *Solanum nigrum*, *Aeschynomene indica*, and *Xanthium strumarium*. The leaf area damage through phytotoxicity of strain-329 increased significantly with increasing the concentration level. The highest phytotoxicity effect was found at the 1x concentration of fermentation medium in both grass and broad leaf weeds. The most apparent effect of the treatment was observed in *Xanthium strumarium* with a necrotic area of 5.1 mm^2^ (Fig 6).

**Fig 7:**
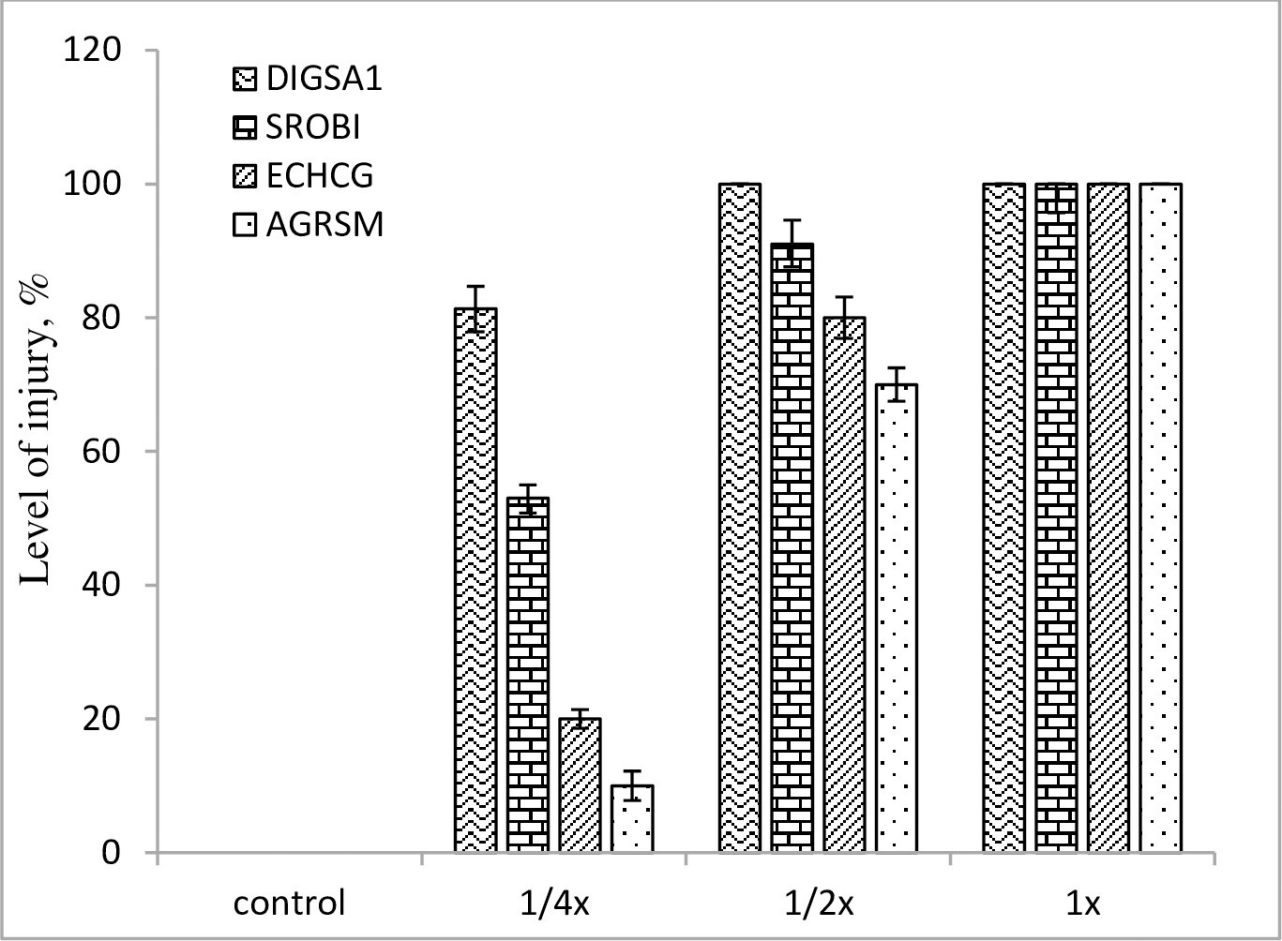
Level of injury in grass weeds at post-emergence application with *Streptomyces anulatus* strain-329 at 14 days after treatment under greenhouse condition.

**Fig 8:**
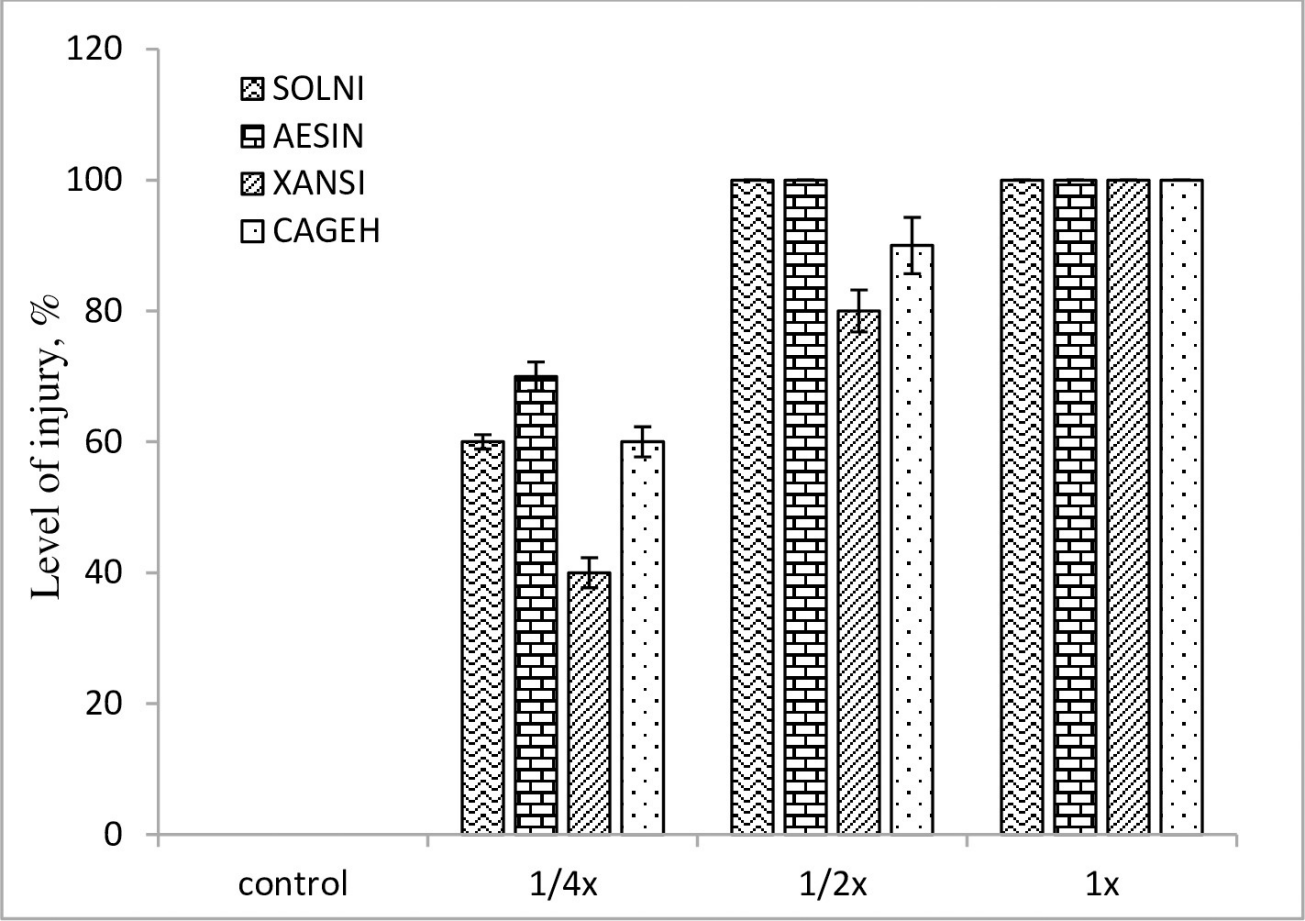
Level of injury in broadleafweeds at post-emergence application with *Streptomyces anulatus* strain-329 at 14 days after treatment under greenhouse condition.

Visible injuries appeared following the fermentation medium treatment of *Streptomyces* strain-329, at the highest concentration with the 1x, the level of injury was 100% in both grass and broadleaf weeds, leading to the plant death (Figs 7 and 8). The bacterial strain showed herbicidal activity mainly on the shoots of the weeds, the effect was more apparent in the broad leaf weeds than that in the grass weeds.

### Evaluation of bioherbicidal activity

*Streptomyces* active compounds coded as 329-C1 and 329-C3 showed strong herbicidal action on treated weed species compared with Cycloheximide (Table 1). However, the compound 329-C3 showed higher phytotoxic injury compared with 329-C1 and the impact was higher with increasing concentrations.

**Table 1:** Herbicidal activity of active compounds 329-C1 and 329-C3 in comparison with Cycloheximide.

Weed species	329-C1 (μg ml^-1^)				329-C3 (μg ml^-1^)				CH (μg ml^-1^)			
	62.5	125	250	500	62.5	125	250	500	62.5	125	250	500
Grass weeds												
DIGSA	20.2a	40.3a	80.2a	100.0a	80.5a	90.2a	100.0a	100.0a	0a	0a	52.1a	80.6b
SROBI	10.5b	30.1b	52.7b	80.2b	60.3b	50.2c	90.1b	100.0a	0a	0a	50.4a	90.3a
ECHCG	10.4b	20.9c	40.1c	50.3c	20.2c	50.5c	100.0a	100.0a	0a	0a	40.2b	80.6b
AGRSM	0c	20.1c	50.3b	50.1c	10.1d	60.1b	100.0a	100.0a	0a	0a	50.4a	80.2b
Broadleaf weeds												
SOLNI	40.2b	40.1c	55.4b	80.5c	50.4b	100.0a	100.0a	100.0a	0a	0a	50.5a	80.4c
AESIN	50.2a	60.4a	74.6a	90.4a	60.3a	100.0a	100.0a	100.0a	0a	0a	54.5a	94.2a
XANSI	30.3c	40.3c	50.6c	50.9d	50.3b	90.3b	100.0a	100.0a	0a	0a	43.6b	83.5b
CAGEH	10.5d	52.2b	62.3b	90.3b	52.4b	90.2b	100.0a	100.0a	0a	0a	52.3a	83.2b

Values with different letters within the same column differ significantly (P<0.05)

C1; 329-C1, C3; 329-C3, CH; Cycloheximide

DIGSA: *Digitaria sanguinalis*, SROBI: *Sorghum bicolor*, ECHCG: *Echinochloa crus-galli*, AGRSM: *Agropyron smithii*, SOLNI: *Solanum nigrum*, AESIN: *Aeschynomene indica*, XANSI: *Xanthium strumarium*, CAGEH: *Calystegia japonica*

The significant growth reductions (100%) in weed biotypes were observed in the production of phytotoxin of active compounds 329-C3 at 500 μg mL^-1^ as compared with the control of 329-C1. Also, at 250 and 500 μg mL^-1^, active compound 329-C3 controlled completely the growth of weed biotypes while the substance 329-C1 showed a growth reduction by 80% in *D*. *sanguinalis*. At 125 μg mL^-1^ concentration, the phytotoxic active compound 329-C3 exhibited a reduction by 90.2% in *D*. *sanguinalis*, *X*. *strumarium*, *C*. *japonica*, and 100% suppressed *S*. *nigrum* and *A*. *indica*, whereas, active compound 329-C1 exhibited a 22–61% growth reduction of grass and broad leaf weed species. At 62.5 μg mL^-1^, maximum reduction of growth (>80%) was found in the active compounds 329-C3 which showed 81.1% killing effect in *D*. *sanguinalis*.

### Purification and structural identification of herbicidal compound

**Fig 9:**
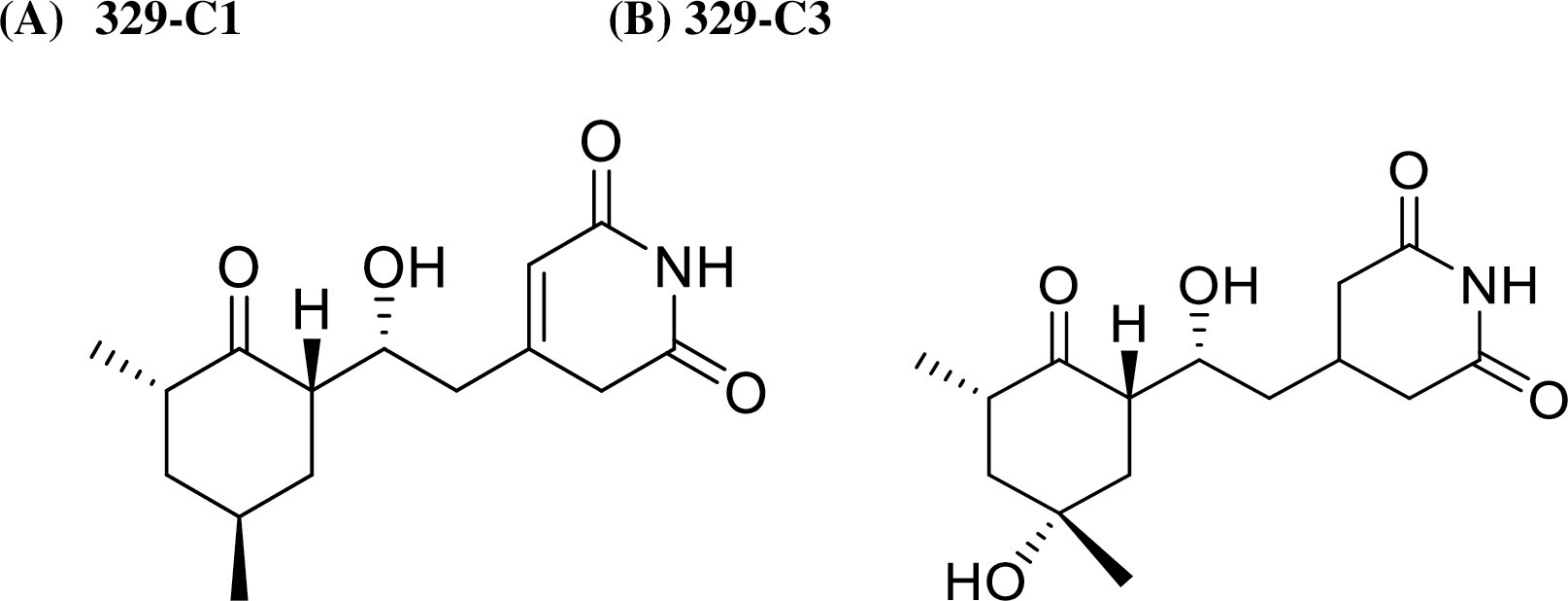
Chemical structural of active compounds 329-C1 (A) and 329-C3 (B) from *Streptomyces* sp. KRA 14–329.

Two herbicidal compounds coded as 329-C1 and 329-C3 were isolated from the liquid culture broth of *Streptomyces* strain-329 by extraction, fractionation and chromatography (Sephadex LH-20). The compounds structures of 329-C1 and 329-C3 were elucidated from an intensive interpretation of ^1^H-NMR, ^13^C-NMR, ^1^H-^1^H COSY, HSQC, and HMBC (800 MHz) in DMSO-*d*_6_. Their molecular formula were confirmed by HR-ESI-TOF Mass spectral data: [M+H]^+^ *m/z* 282.1700 (calcd, 282.1705 for C_15_H_23_NO_4_+H) and [M+Na]^+^ *m/z* 304.1523 (calcd, 304.1525 C_15_H_23_NO_4_+Na) for 329-C1 with a molecular weight of 281.2 and [M+Na]^+^ *m/z* 320.1471 (calcd, 320.1474 for C_15_H_23_NO_5_+Na) for 329-C3 with a molecular weight of 297.2 (Fig 9).

### Effect on chlorophyll loss

**Fig 10:**
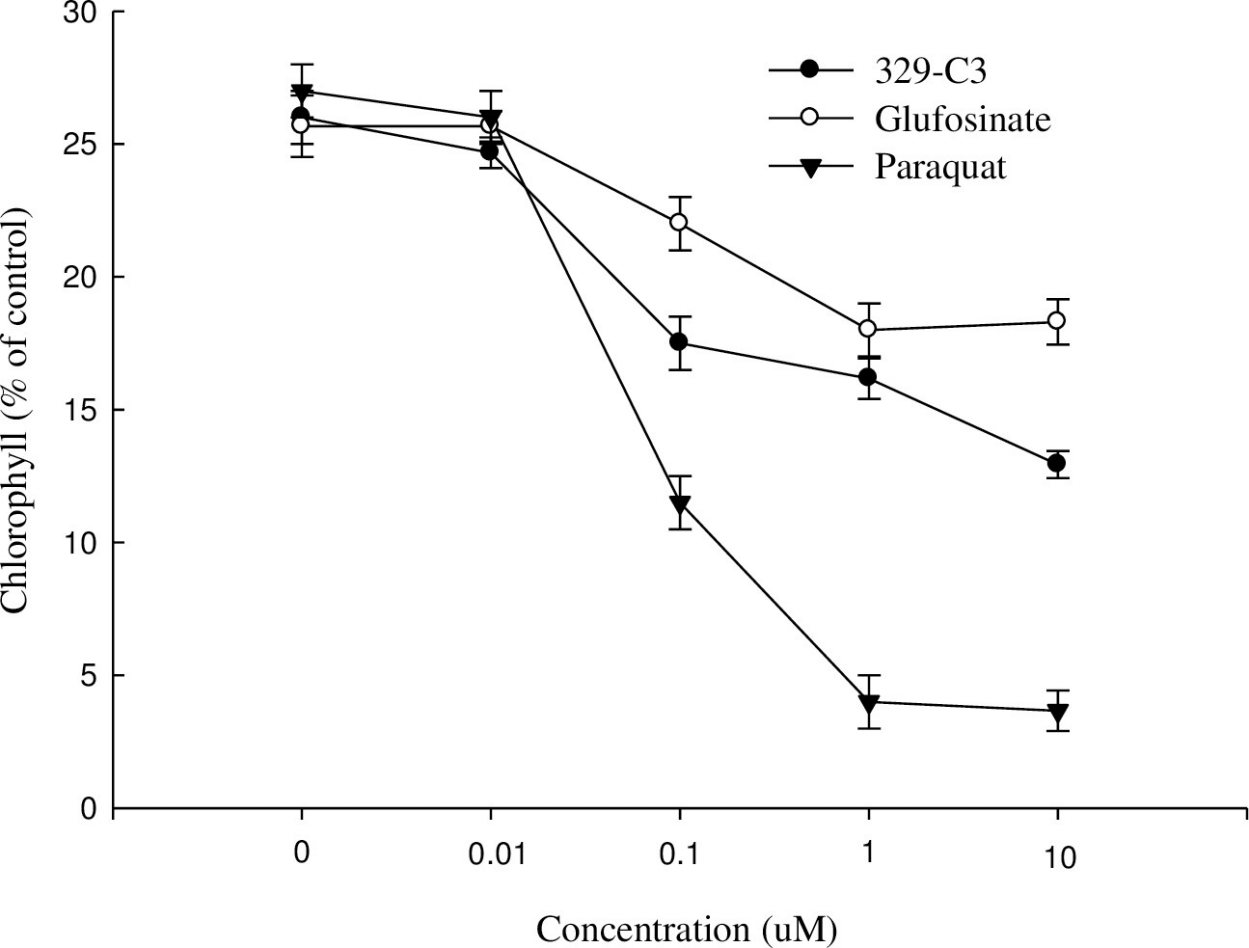
Reduction of chlorophyll in cucumber by paraquat, glufosinate-ammonium, and active compounds 329-C3 at different concentrations. The tissue was exposed to continuous light at 120 μmol m^-2^ sec-^1^ PAR at 25°C following 12 h of dark incubation. Error bars are ±1 SE of the means.

Effect on chlorophyll loss was evaluated by paraquat, glufosinate-ammonium, and active compound 329-C3 in cucumber leaf disks. In this assay, the chlorophyll content decreased with increasing concentration of the solutions. The concentrations of 0.1, 1, and 10 μM showed adverse chlorophyll loss in paraquat treatment compared with glufosinate-ammonium and active compound 329-C3 (Fig 10).

In the chlorophyll reduction process, paraquat decreased by 88.9% chlorophyll content compared to untreated leaf disks in a concentration of 10 μM, whereas, active compound 329-C3 and glufosinate-ammonium reduced about 50.4% and 31.2% chlorophyll content, respectively.

### Influence on the electrolyte leakage

**Fig 11:**
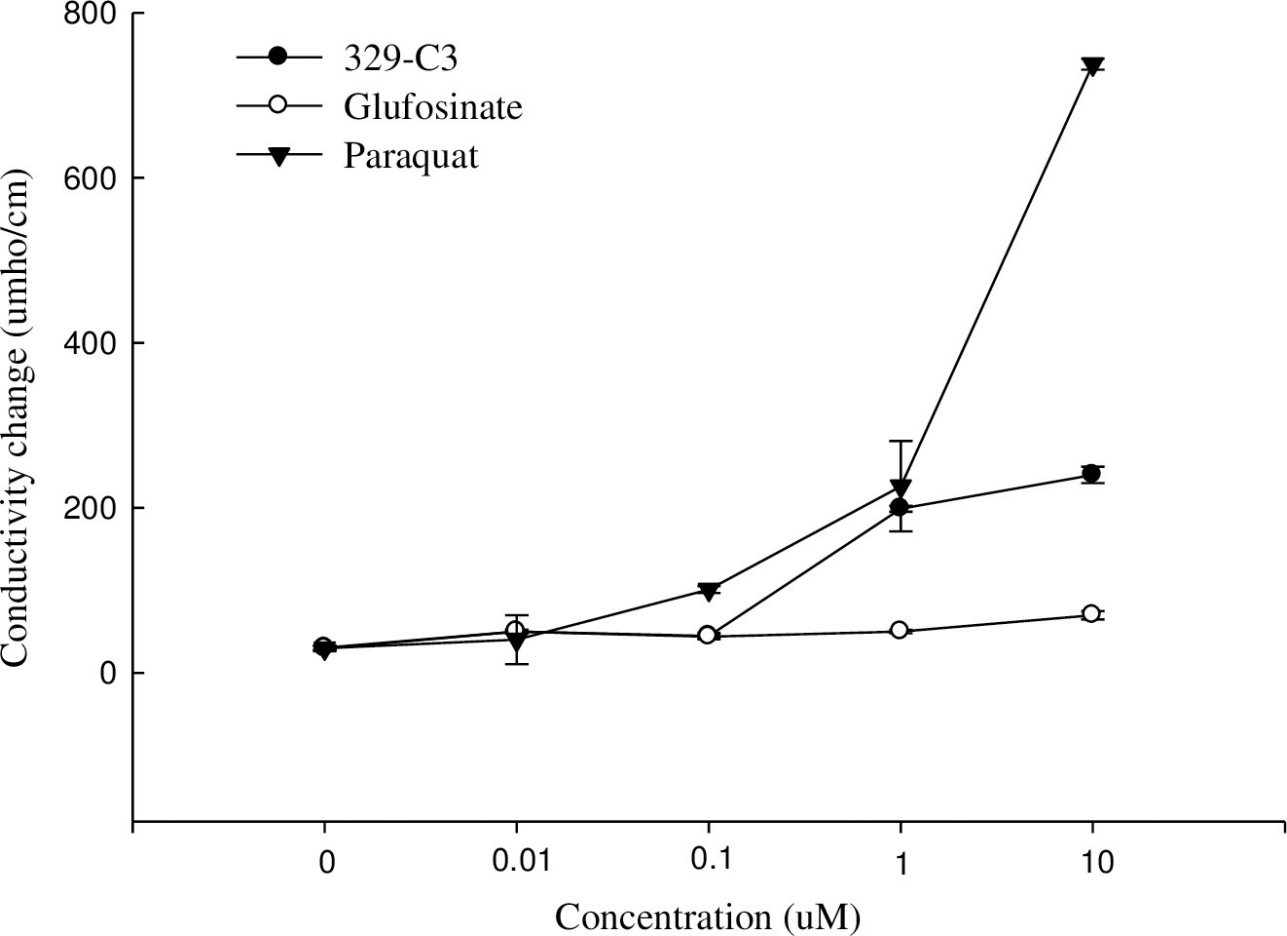
Electrolyte leakage in cucumber by paraquat, glufosinate-ammonium, and active compound 329-C3 at different concentrations. Error bars are ±1 SE of the means.

There was a significant difference in the degree of electrolyte leakage substances between the herbicidal active compound 329-C3 and two control agents, paraquat and glufosinate-ammonium (Fig 11).

As a result of measuring the degree of change in conductivity after 12 h of light irradiation, there was almost no difference between the amount of electrolyte leakage in the untreated case and the leakages on all concentrations of glufosinate-ammonium solution. In the case of active compound 329-C3, the amount of electrolytic leakage raised with increasing concentration, but the change in conductivity was 220 μmho cm^-1^ which was lower than that of paraquat, 780 μmho cm^-1^ at the 10 μM concentration.

### Effect on lipid peroxidation

**Fig 12:**
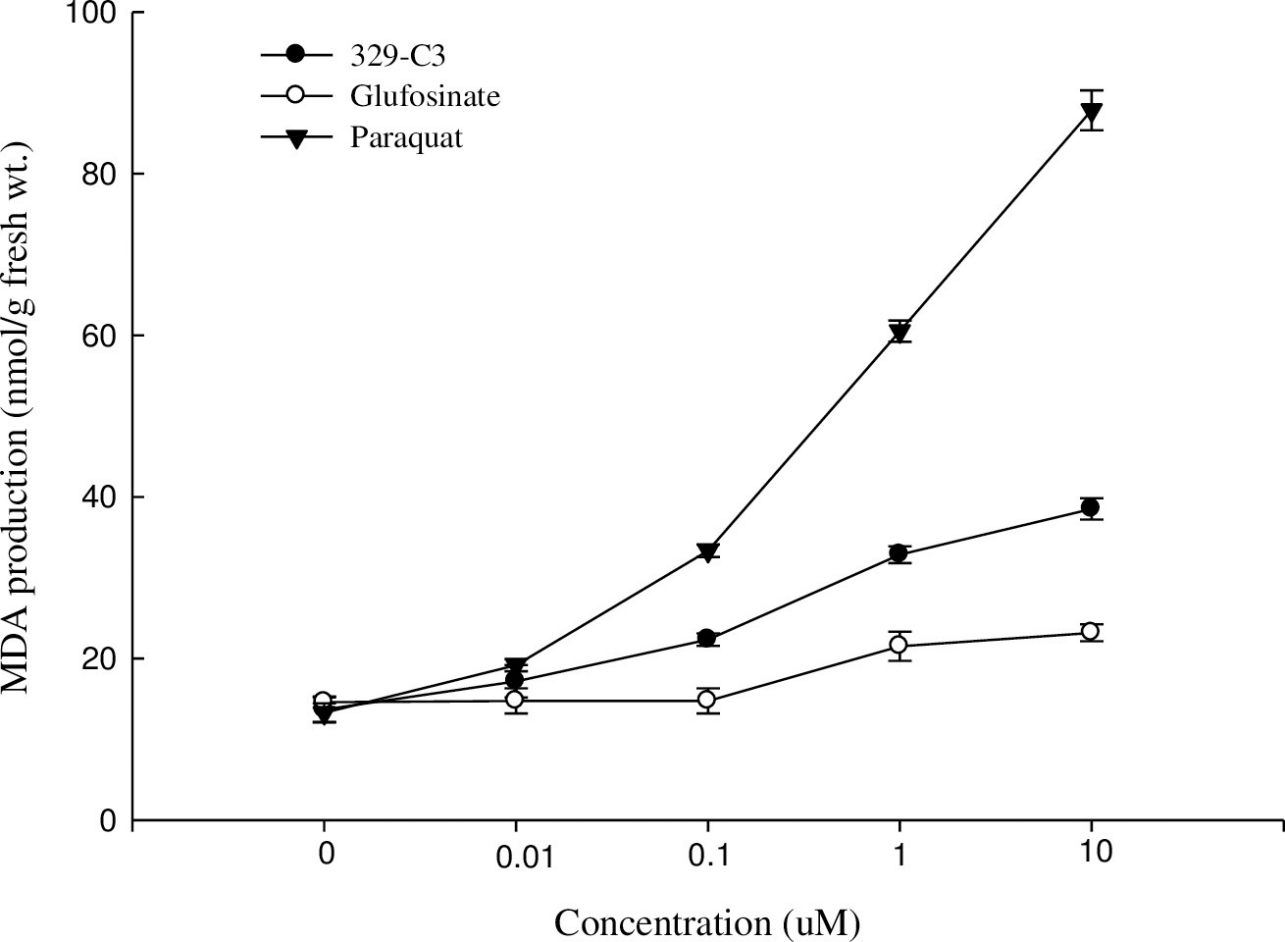
Effect of paraquat, glufosinate-ammonium and active compound 329-C3 on MDA production in cucumber leaves. Error bars are ±1 SE of the means.

The level of MDA production was determined by the estimation of cell membrane lipid peroxidation of cucumber leaf disks following the treatments with paraquat, glufosinate-ammonium, and active compound 329-C3. The MDA production of 329-C3 was relatively low as compared with paraquat and higher than that of glufosinate-ammonium with increasing concentrations (Fig 12).

In the initial concentration, 0.01 μM, the amount of MDA in paraquat, glufosinate-ammonium, and 329-C3 were 20.8, 15, and 19.2 nmol, respectively. Whereas, the amounts of MDA were 95, 24.8, and 43 nmol in paraquat, glufosinate-ammonium, and 329-C3, respectively at 10μM concentration. An increasing trend in the MDA production occurred with an increase of the concentrations greater than 0.01 μM.

## Discussion

Biological control of weeds using soil microorganisms is an essential alternative to a chemical treatment to reduce weed populations and their harmful effects. The present study revealed that *Streptomyces* strains can be found ubiquitously in soil samples. The isolated *Streptomyces* strains were evaluated on phytotoxic activity, aiming a discovery of bioactive compounds with herbicidal action. Only 8% of the isolated strains showed some level of herbicidal effect on the seedling growth of *D*. *sanguinalis*. The experiments conducted by DeFrank and Putnam [26] were in agreement with our results, where 7% of 120 *Streptomyces* isolates inhibited 60% of germination and growth of barnyardgrass and cucumber (*Cucumis sativus* L.). Also, Heisey et al. [27] expressed that 10–12% of 347 *Streptomyces* isolates from their testing on solid medium showed highly inhibitory to three indicator seedlings.

Phytotoxic effects of strain-101 and strain-128 may not necessarily cause the same effect on weeds as strain-329 because each bioherbicide has specific function through infection or toxification of weed cells. On the other hand, weed species also differ to each other on the sensitivity to control treatments. *Agropyron smithii* and *Xanthium strumarium* belonging to grass weed and broadleaf weed families, respectively were tolerant to the treatment with the fermentation medium of strain-329. Sensitivity experiments of the weeds to strain-329 showed that *D*.*sanguinalis* belonging to the grass weeds family and *Solanum nigrum* and *Calystegia japonica* belonging to the broadleaf weeds family were higher sensitive at the pre-emergence application of strain-329.

At the post-emergence application, a significant growth reduction from 70 to 100% was recorded in all treated weeds species with the fermentation medium of *Streptomyces* strain-329 at 1x and 1/2x concentrations (Figs 7 and 8). However, the highest growth reduction (80%) was achieved in *D*. *sanguinalis* at the 1/4x concentration as compared with untreated control. The level of suppression in the weeds increased with increasing concentration of the fermentation medium. The study conducted by El-Sayed et al. [28] also found variable effect of natural herbicidal products derived from the culture broth of *Streptomyces* spp.

The phytotoxic activity of strain-329 at the 1x concentration had substantial inhibition effect on the growth of the weeds and caused visual injury symptoms, demonstrating high efficiency at post-emergence application. Thus, the highest phytotoxicity rate (100%) was observed at the 1x concentration of fermentation medium for both grass and broadleaf weed species. Sondhia and Saxena [29]also found that similar effect of herbicidal extracts of actinomycetes on the germination and seedling growth of *E*. *crusgalli*. The phytotoxic activity indicates that lower concentration had a functional activity to the susceptibility of each weed species. The inhibitory effects of microbial isolates towards the target weeds are due to the presence of phytotoxic compounds. The mode of action of bioherbicides is similar as plant-pathogen interaction mechanisms [30]. In the case of the plant-pathogen interaction, phytopathogen degrades cell wall, proteins, and lipid membrane of the plant and spread onto the host plant [31]. Also, phytopathogens could generate phytotoxic secondary metabolites and peptides that form toxins which interfere with plant metabolism [32]. These toxins directly or indirectly affect the expression of genes and eventually lead to plant death [33]. Walton [34] described that pathogens can interfere in metabolism processes of a plant by producing some extracellular agent or toxin that affects to meristematic cells. Such activity was more visible after the application of strain-329 in this experiment, which provided greatest damages, such as yellowing, followed by sharp wilting and complete collapse of *D*. *sanguinalis* growth. Yandoc et al. [35]also informed the effects of some pathogenic fungus inducing leaf spot, yellowing and plant death of *Imperata cylindrica*. While studying the effect of bioherbicides, Hoagland et al. [36] found that susceptibility of each weed species varied according to bioherbicidal activity, on the other hand, phytotoxic metabolites also differ in functioning on target-specificity.

*Streptomyces* are characterized by colony formation, vegetation and aerial mycelium which are the most important morphological characteristics [37, 38]. The characteristics of natural herbicidal active compounds showed contact type, non-selective herbicidal effect, and burn-down symptoms on treated leaves or stems of plants [39]. In addition, natural herbicides have no any residual effects in a certain period of application [40]. In this study, the *Streptomyces* strain-329 (329-C1 and 329-C3) presented broad herbicidal activities against both grass and broadleaf weed species. As shown in Table 1, the herbicidal activity of the *Streptomyces* compounds was apparent, especially against broadleaf weeds. The active compound 329-C3 had a more selective effect in both grass and broadleaf weeds than the 329-C1.

Chlorophyll loss and membrane lipid peroxidation are typical indicators of phytotoxicity for active compounds of bioherbicide. According to the degree of change in chlorophyll, paraquat and the herbicidal active compound 329-C3 were able to confirm a significant effect to prevent secondary metabolites. An herbicide which inhibits photosynthesis, shows herbicidal action, blocks electron transfer in PSII or destroys cells by taking electrons from PSII. Such herbicides cause a sharp decrease in a chlorophyll content of leaves after treatment [40, 41]. These results showed that a cell membrane was destroyed over time after the treatments. However, in the case of paraquat, the amount of electrolytic leakage was the highest after 12 h of light irradiation since it had a mechanical action that destroyed the cell membrane directly to generate reactive oxygen. In the glufosinate-ammonium treatment, there was no leakage of electrolytic material because this herbicide exhibits the mechanism to kill plants by ammonia poisoning action produced by inhibiting glutamine biosynthesis process to destruct a cell membrane. The active compound 329-C3, in the case of electrolytic leakage, was intermediate between paraquat and glufosinate-ammonium, but it was significantly lower than that of paraquat. On the other hand, the compound 329-C3 may have another mechanism of action than the inhibition of glutamine biosynthesis.

Based on the above results, the *Streptomyce* substance 329-C3 is not a major photosynthesis inhibitor such as paraquat, which has a main function in the plant body, and presumed to exert an another mechanism than inhibition of glutamine biosynthesis such as glufosinate-ammonium.

## Conclusion

Among initial selected 102 isolates out of 1,300 bacterial strains, only three bacterial strains showed considerable phytotoxicity, but apparent herbicidal effect was achieved with *Streptomyces anulatus* strain-329. A positive correlation was observed between the phytotoxic activity and the concentrations of *Streptomyces anulatus* strain-329 at both pre and post-emergence applications in the tested weeds species. The organic extract of the *Streptomyces anulatus* strain-329 exhibited desirable bioherbicidal traits such as high virulence and broad weed host range and could be used as a bioherbicide for weed control.

Results confirmed that the *Streptomyces* strain-329 contains a natural active compound to have contact and selective activity, especially target-specific toxicity on the selected weed plants. The *Streptomyces* strain-329 producing herbicidal metabolites may provide a new molecule for a more efficient herbicidal effect to control of weeds. Further study will be required to confirm an herbicidal activity in experimental trials in a field to elucidate herbicidal action and mechanisms.

## Supporting information

S1 Fig Screening herbicidal activity of soil *Streptomyces* on the plant growth of *Digitaria sanguinalis* at 7 days after foliar application under the greenhouse condition. (TIF)
