# Supplementary figures and images for: Isolation, identification and characterization of Streptomyces metabolites as a potential bioherbicide
Source: PLoS One. 2019 Sep 23;14(9):e0222933. doi: 10.1371/journal.pone.0222933 (PMC6756554; doi:10.1371/journal.pone.0222933)

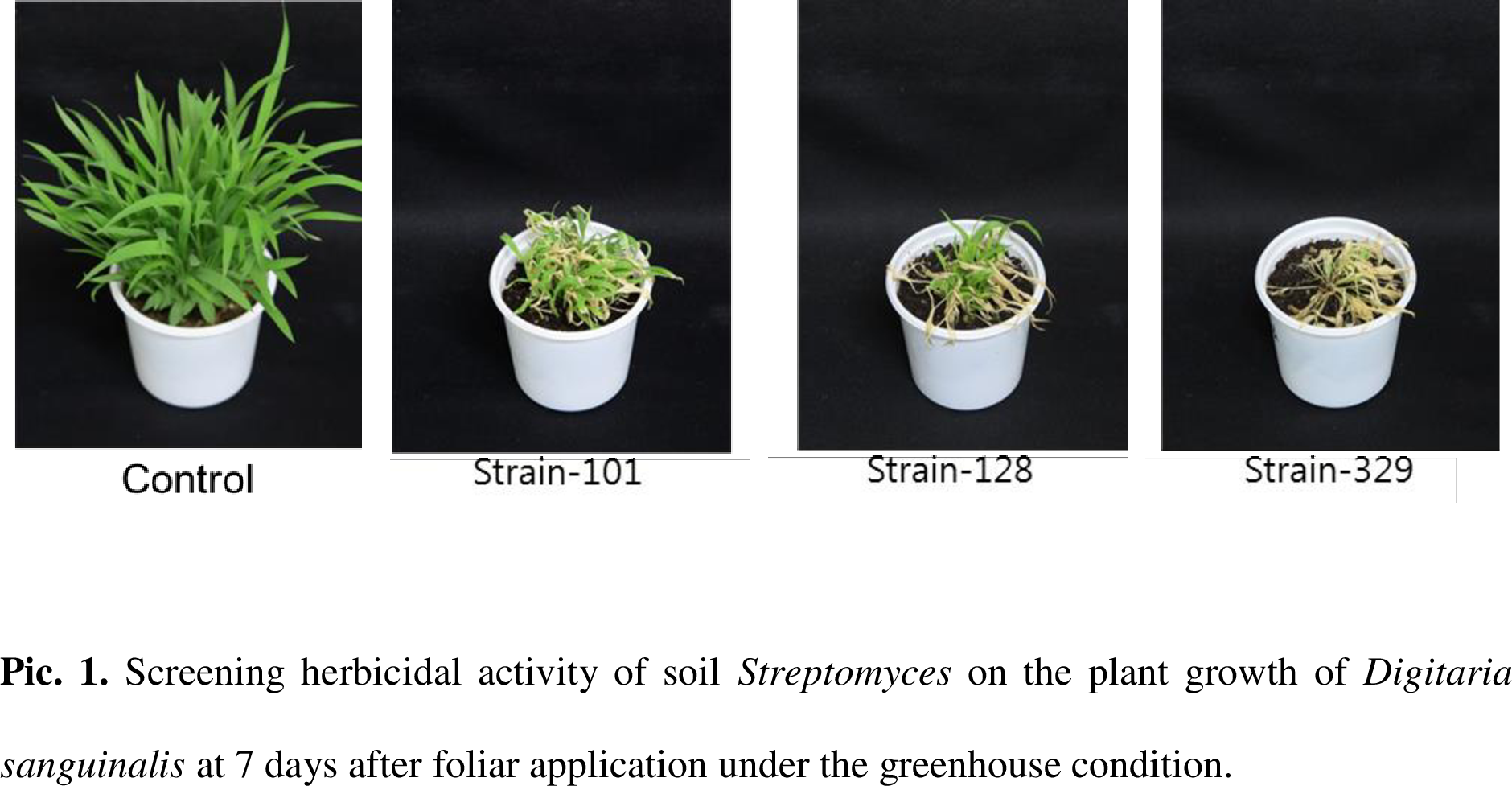

Supplement: S1 Fig — (TIF) [file pone.0222933.s001.tif]
